# Psychiatric admissions from the emergency department: An observational, retrospective study and recommendations for improved patient care and use of resources

**DOI:** 10.1192/j.eurpsy.2021.972

**Published:** 2021-08-13

**Authors:** J. Ellul Pirotta, E. Saliba, M.R. Cassar

**Affiliations:** 1 Emergency Department, Mater Dei Hospital, Msida, Malta; 2 Psychiatry, Mount Carmel Hospital, Attard, Malta

**Keywords:** Medical Clearance, emergency, Investigations, diagnosis

## Abstract

**Introduction:**

Psychiatric patients visiting the Emergency Department (ED) often require ‘medical clearance’. We aim to review patient work-up in the ED to facilitate the management of these patients.

**Objectives:**

- To identify common demographic variables, diagnoses and mental health legislative status of patients presenting to the ED requiring psychiatric admission - To assess whether patients underwent a medical work-up in the ED, and what investigations were carried out - To produce a hospital proforma for the management of psychiatric patients presenting at the ED

**Methods:**

Data on adult psychiatric patients visiting the ED over a six month period was collected retrospectively, which was then analysed accordingly.

**Results:**

473 patient admissions were reviewed. 32.8% were admitted to a non-psychiatric specialty before being accepted to psychiatry, with the most common reasons being due to overdose (30.3%), alcohol-related problems (19.4%), and medical complaints (18.7%). 63.2% of all patients were investigated in the ED, including 23.5% undergoing CT Brain imaging. The majority had a final diagnosis falling under F10-19 (30.2%) and F30-39 (30.9%) chapter categories of the ICD-10, with the former having the highest absolute number of patients undergoing testing in the ED. The F20-29 group (13.7%) was highest in total patients investigated (75.4%), CT brain imaging (56.9%), and rate of involuntary admissions (33.8%), suggesting they are the most resource intensive group.

**Conclusions:**

Patients with acute mental disorders present significant challenges to emergency physicians. Staff education and an inter-departmentally agreed upon proforma, taking into account the results of this study, may facilitate management of these patients within the ED.

